# The Importance of Latinx Cultural Values in Caregiving in Rural Communities

**DOI:** 10.1093/geroni/igaf122.833

**Published:** 2025-12-31

**Authors:** Karen Kopera-Frye

**Affiliations:** New Mexico State University, Las Cruces, New Mexico, United States

## Abstract

CDC reports almost 23% of adults are caregivers to family or friends in New Mexico; 10.4 % provide care to an individual with dementia. The southern Border region of New Mexico is remote and desert, with high rates of poverty and inaccessibility to health care and services. The incidence of Latinx caregivers is rising in light of the increasing population trends. Yet, available culturally appropriate support services are lacking. Familismo, a Latinx primary cultural value, refers to the importance of strong family loyalty, closeness, and getting along with and contributing to the well-being of the nuclear family, extended family, and kinship networks. This value is additionally responsible for the increased caregiving seen among many Latinx families. The family is extremely important, yet appropriate services and resources may not be available, causing distress. Seventy-six primarily female Latinx caregivers were surveyed on caregiving they provide, perceptions of preparedness, needs, positive and negative aspects of caregiving. Results indicated the top three provided aspects of caregiving were (in descending order): Food, emotional support, and transportation. Caregivers reported a high degree of preparedness, e.g., taking care of the recipient’s physical needs. Needed resources included emotional support, financial assistance, and respite. Greater caregiving provision was significantly associated with greater resource needs (r = .35, p <.01). Despite multiple needs, caregiving perceptions were largely positive, highlighting the importance of the cultural value of familismo in Latinx families. Having culturally responsive service providers can decrease some of the challenges such as health disparities in rural communities.

